# Ritgen’s maneuver in childbirth care: A case-control study in a Central Italian setting

**DOI:** 10.18332/ejm/192698

**Published:** 2024-11-01

**Authors:** Simona Salusest, Silvia Salvi, Federica Totaro Aprile, Ada Rubini, Francesca Stollagli, Silvia Buongiorno, Roberta Rullo, Jessica Preziosi, Gloria Anderson, Michelangela Danza, Antonio Lanzone

**Affiliations:** 1 UOC di Ostetricia e Patologia Ostetrica, Dipartimento di Scienze della Salute della Donna e del Bambino e di Sanità Pubblica, Fondazione Policlinico Universitario ‘A. Gemelli’, IRCCS, Roma, Italia; 2 Università Cattolica del Sacro Cuore, Roma, Italia

**Keywords:** manual perineal protection, Ritgen’s maneuver, perineum, birth trauma, maternal outcome

## Abstract

**INTRODUCTION:**

Vaginal delivery can cause genital tract trauma and lacerations of different severity. This study aims to establish if routinary use of Ritgen’s maneuver decreases the prevalence and severity of perineal lacerations compared to the traditional manual perineal protection (MPP).

**METHODS:**

This prospective case-control study was conducted in the labor ward of Fondazione Policlinico A. Gemelli, Rome, Italy. One hundred sixteen women who met inclusion criteria were included. The study group (n=58) consisted of women who were assisted using the Ritgen maneuver, whereas the women who gave birth immediately afterward were selected as the control group (n=58). All information was retrieved through electronic medical records.

**RESULTS:**

In all, 22% women of the study group reported no perineal lacerations compared with 5% of the control group (p=0.007). Regarding the degree of lacerations, the study group exhibited more first-degree lacerations and fewer second-degree lacerations, while the control group exhibited the opposite trend. Among women who received epidural analgesia, 24% of the study group did not experience perineal lacerations, compared to 4.5% of the control (OR=0.15; 95% CI: 0.03–0.72; p=0.008). Similarly, 23.4% of cases in the study group with oxytocin-enhanced labor, experienced no perineal trauma while none in the control group had no perineal trauma in cases of oxytocin augmentation (p=0.005).

**CONCLUSIONS:**

Our results suggest that using Ritgen’s maneuver in childbirth care may reduce the incidence and severity of perineal lacerations, even in the presence of known risk factors for perineal lacerations such as oxytocin augmentation and epidural analgesia.

## INTRODUCTION

Vaginal delivery is worldwide associated with genital tract trauma in 85% of women^1-3^. Perineal trauma is defined as any type of damage to female genitalia during childbirth, which may occur spontaneously or iatrogenic (via episiotomy or instrumental delivery). It can be classified as anterior perineal trauma when it affects the anterior vaginal wall, urethra, clitoris, and labia, or posterior perineal trauma, the most frequently observed during childbirth, that is, any injury to the posterior vaginal wall, perineal muscles, or anal sphincter^1,2,4^. Moreover, this is further classified as a first-degree tears when they involve the perineal skin only; second-degree tears involve the perineal muscles and skin; third-degree tears involve the anal sphincter complex (classified as 3a where <50% of the external anal sphincter is torn; 3b where >50% of the external anal sphincter is torn; 3c where the internal and external anal sphincter is torn); and fourth-degree tears involve the anal sphincter complex and anal epithelium^4^.

It is well established that perineal tears can negatively affect maternal recovery in the immediate postpartum period and have a physical and psychological impact on women’s health in both the short- and long-term, mainly for its consequences in terms of anal incontinence, perineal pain, and dyspareunia^2,5^. Risk factors associated with perineal tears can be distinguished in maternal conditions (nulliparity, Asian ethnicity, vaginal birth after cesarean, maternal age ≤20 years, perineal body length <25 mm), fetal conditions (fetal weight >4000 g, occiput posterior positions) and intrapartum conditions (operative vaginal delivery, prolonged second stage of labor, epidural analgesia, oxytocin augmentation, lithotomy, and supine position)^6^.

Different perineal protection techniques are used to slow the expulsion of the fetal head and to allow the perineum to stretch slowly to prevent trauma. The World Health Organization (WHO), in ‘Intrapartum Care for a Positive Childbirth Experience’, recommends, in the second stage of labor, different techniques to reduce perineal trauma, including perineal massage, warm compresses, and manual perineal protection (MPP)^7^. The evidence is derived from a Cochrane systematic review with twenty trials involving 15181 women who contributed data^5^. In this review, two studies evaluating Ritgen’s maneuvers have considered and suggested that Ritgen’s maneuver may have little or no impact on third- and fourth-degree perineal tears and episiotomy^5,8,9^.

The National Institute for Health and Care Excellence (NICE) Intrapartum Care guidelines find no difference between hands-poised and hands-on techniques in the prevention of obstetrical anal sphincter injuries (OASI)^10^. According to the Royal College of Obstetricians and Gynaecologists (RCOG) there is no robust evidence to state which is the best method of perineal support/protection during the expulsive period of the second stage of labor with the Ritgen maneuver no better than perineal protection/hands-on^4^.

However, in a context where there is no sufficient evidence to establish which is the best technique for MPP, this study aims to compare the prevalence and severity of perineal tears between women assisted with the Ritgen maneuver in comparison to women assisted with conventional manual perineal protection, during the expulsive period of the second stage of labor, in an Italian Central setting.

### Country-specific background

The Italian healthcare system offers universal and free-of-charge maternity care. Almost all births in Italy take place in hospital^11^. The National Health System midwives work in the community or in the hospital, where they rotate between labor areas, antenatal and postnatal wards. In Italy, no national intrapartum guidelines exist leading to a very heterogeneous panorama regarding midwifery practice during labor and delivery among institutions, with huge variations between regions^11,12^. Data from the National Outcomes Evaluation Program (2015–2020) found, for example, a strong decrease in episiotomies in vaginal deliveries (from 24.0% to 13.8%), but with a strong North-South gradient in Italy^13^. There are no national data regarding the use of epidural analgesia and oxytocin augmentation.

## METHODS

### Study design and setting

This is a prospective case-control study conducted in the Fondazione Policlinico Universitario Agostino Gemelli IRCCS delivery unit in Rome, Italy. Data collected covered the period from May 2020 to October 2021.

### Participants

A total of 116 women were included in the study. Inclusion criteria were singleton pregnancy, gestational age >37 weeks, cephalic presentation, nulliparity, neonatal weight between the 10th and 90th percentile, maternal age 20–40 years, spontaneous labor or not more than one attempt of pharmacological induction; all women who underwent operative vaginal delivery and episiotomy were excluded. The study group (n=58) consisted of women who were assisted using the Ritgen maneuver. For each woman assisted using Ritgen’s maneuver, a woman who gave birth immediately afterward was selected as a control if she was assisted according to conventional practice and met the inclusion criteria. This group constituted the control group (n=58).

**Figure 1:**
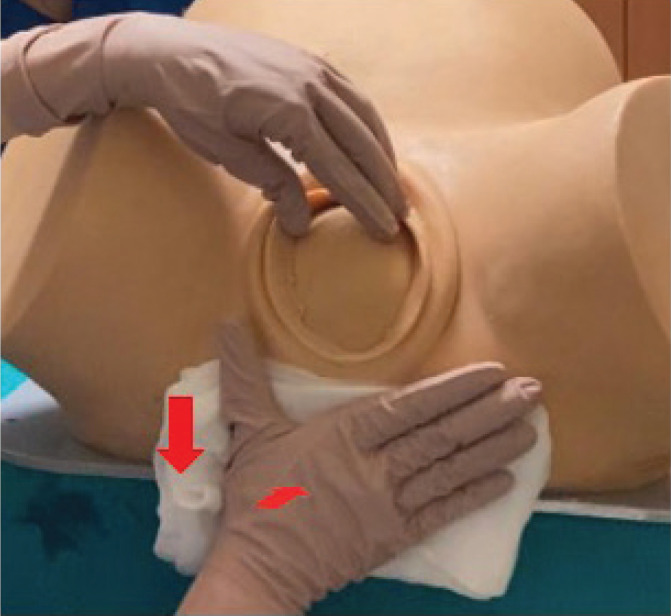
Manual perineal protection performed reproducing the Viennese technique

Controlled women were assisted using the standardized manual perineal protection in use in our center that reproduced the Viennese technique and was performed as follows: when the presenting part began to emerge and was close to crowning, the midwife’s dominant hand, covered by gauze, was placed flat to protect the posterior perineum. The left hand was then placed in contact with the two fetal parietal bones (when the head had exited 3–4 cm from the vulvar rim), with the thumb on one side and the index and middle fingers on the other. This was done without imposing resistance but simply following the progression of the presenting part (Figure 1).

**Figure 2:**
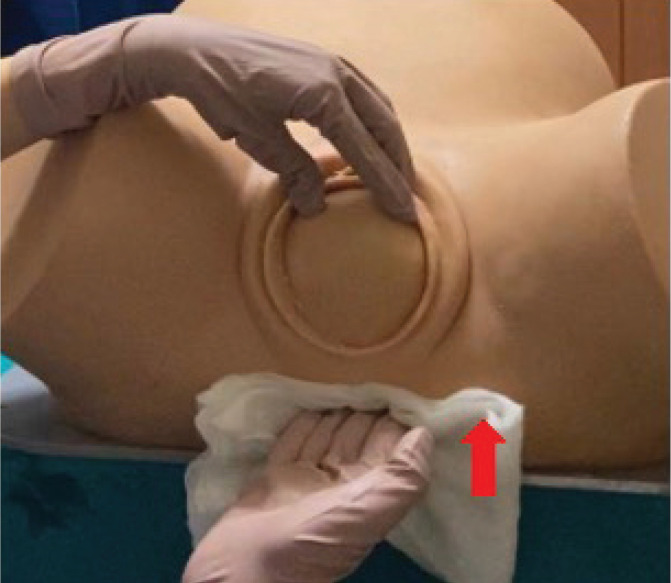
Ritgen’s maneuver perineal protection procedure

The Ritgen maneuver was performed during the uterine contractions, as described by Habek et al.^14^ and Jönsson et al.^9^, and according to the following principles: the fingers of the dominant hand were pointed towards the middle of the perineum, exerting pressure from bottom to top. Meanwhile, the non-dominant hand’s two or three fingers (usually the thumb, index, and middle finger) were placed in contact with the fetal occiput to accompany its deflection, thus delaying it (Figure 2).

The outcomes investigated were the incidence and the severity of perineal tears. In defining perineal trauma, the classification established by Sultan et al.^15^ and adopted by the International Consultation on Incontinence and RCOG^4^ was used. The perineum is defined as intact when it is such in all its components (muscles, fascial tissue, erectile bodies, ligaments, nerves, sphincters). The perineum is defined as lacerated when, following the passage of the fetus, trauma is created, more or less extensive, more or less visible, with involvement of one or more tissue layers. The lacerations can compromise the anatomical integrity, which can be evidenced and repaired immediately after childbirth, or the functional integrity, which is delayed in time. The recovery is slow and sometimes possible only by surgery. The perineal lacerations immediately detectable after childbirth are classified according to the depth and the tissues involved. We can distinguish lacerations of the anterior perineum, which are less frequent, involving the nymphs and/or para-clitoral region, and of the posterior perineum, which occur more often. Among the posterior lacerations there are:

- First-degree: when they affect the vaginal mucosa but not the perineal muscles;
- Second-degree: when they involve both the mucosa and the perineal muscles;
- Third-degree: when in addition to the vaginal mucosa and perineal muscles, affect some or all of the fibers of the anal sphincter; and
- Fourth-degree: when they also affect the rectal muscularis^16^.

### Variables

All information related to the woman (maternal age, ethnicity, pre-conceptional body mass index, comorbidities), the newborn (sex, birth weight and percentile, Apgar score at 1st and 5th minutes), and delivery (gestational age, oxytocin use, induction method, use of epidural analgesia, blood loss, fetal head position, second stage duration, incidence and degree of perineal laceration) were retrieved through the electronic medical records, contained in the Trackcare Unified Healthcare Information System and Digistat system. Neonatal weight percentile was calculated using the neonatal national standard weight curve developed by Ferrazzani et al.^17^. To calculate the duration of the second stage, the definition recommended by the WHO was used: ‘The second stage of labor is the time between complete dilation and the birth of the baby, during which the woman manifests the involuntary need to push followed by expulsive contractions’ for which the time elapsed between the time when the obstetric visit recording complete dilation was performed and the time of birth of the newborn was calculated^7^.

### Bias

To mitigate bias related to professional experience and technique, a single midwife was assigned to perform this maneuver, ensuring consistency across all women meeting the inclusion criteria during the study period. However, using a single operator may also introduce a systematic bias into the results that we have considered.

### Ethics

The project was approved by the Institutional review board of the Institute of Obstetrics and Gynecology of the Catholic University of the Sacred Heart, Rome where the project has been developed (N° Prot. Aprov. IST CICOG-04-03-19/14). All women gave their consent for participating before the admission to the study and for their clinical data to be collected and analyzed for scientific purpose.

### Statistical analysis

The Kolmogorov-Smirnov and the Shapiro-Wilk tests were used to assess the normality of data distribution. Categorical data are presented as frequencies and percentages, and continuous normally distributed data are presented as mean and standard deviation. Normally distributed continuous data were compared using the t-test non-paired. Cases with missing values were excluded from the analysis. The chi-squared test was used to evaluate the observed frequencies. For women undergoing Ritgen’s maneuver, the odds ratio (OR) with a 95% confidence interval was additionally evaluated for the main outcome of the study, perineal trauma, in relation to two exposures, the use of epidural analgesia and the oxytocin augmentation. A logistic regression was also performed to ascertain the effect of different independent variables (type of MPP, maternal age, labor induction, epidural analgesia, oxytocin augmentation, second stage duration, labor induction) on the likelihood that women have perineal trauma (dependent variable). The software used for the statistical analysis was the Statistical Package for Social Science (SPSS) Version 25. The p-value of each statistical analysis was calculated, and the threshold for significance was set at p<0.05.

## RESULTS

During the study period, 116 women met the inclusion criteria, 58 belonging to the control group (receiving traditional obstetrical care, with MPP according to Viennese technique) and 58 to the study group (receiving Ritgen’s maneuver). Maternal sociodemographic and clinical findings are described in Table 1. Maternal age and ethnicity did not differ between the two groups. Both maternal height and weight were statistically lower in the study group than the control (p=0.014 and p=0.046, respectively); no differences in pre-conceptional BMI and gestational weight gain were observed. In Table 2, neonatal outcomes are illustrated. Gestational age at delivery was similar between groups. A lower percentage of women assisted with Ritgen’s maneuver gave birth to infants weighing between the 50th and 75th percentiles (22.4%) compared to the control group (36.2%). Conversely, neonates weighing between the 25th and 50th percentiles were more common in the study group (48.3%) than in the control (31.0%).

**Table 1:** Maternal sociodemographic and clinical characteristics of the two groups of pregnant women, aged 20–40 years, divided according to the preventing perineal maneuver used in the Fondazione Policlinico Universitario Agostino Gemelli delivery unit, May 2020 – October 2021, Rome, Italy (N=116)

*Characteristics*	*Routine obstetric care (N=58) n (%)*	*Ritgen’s maneuver (N=58) n (%)*	*p^a^*
**Maternal age** (years), mean ± SD	31.81 ± 4.30	31.24 ± 4.91	0.508
**Ethnicity**			
White	55 (94.8)	55 (94.8)	0.549
Black/African	0 (0)	1 (1.7)	
South American	3 (5.2)	2 (3.5)	
**Height** (m), mean ± SD	1.67 ± 0.06	1.64 ± 0.06	0.014
**Weight** (kg), mean ± SD	61.98 ± 9.22	58.51 ± 8.71	0.046
**BMI** (kg/m^2^)			0.711
≤18	0	0	
19–25	33 (56.9)	31 (54.4)	
26–30	18 (31.0)	21 (36.8)	
31–35	6 (10.3)	5 (8.8)	
>35	1 (1.7)	0	
**Gestational weight gain** (kg), mean ± SD	11.79 ± 3.95	12.44 ± 4.27	0.384

BMI: body mass index.

a The p-value has been calculated between women receiving routine obstetric care versus those who underwent Ritgen’s maneuver.

**Table 2:** Neonatal outcome of the two groups of pregnant women, aged 20–40 years, divided according to the preventing perineal maneuver used in the Fondazione Policlinico Universitario Agostino Gemelli delivery unit, May 2020 – October 2021, Rome, Italy (N=116)

*Variables*	*Routine obstetric care (N=58) n (%)*	*Ritgen’s maneuver (N=58) n (%)*	*p^a^*
**Gestational age at delivery** (weeks), mean ± SD	39.46 ± 1.02	39.27 ± 0.92	0.289
**Neonatal weight percentile**			0.237
<25	12 (20.7)	10 (17.2)	
25–50	18 (31.0)	28 (48.3)	
50–75	21 (36.2)	13 (22.4)	
>75	7 (12.1)	7 (12.1)	
**Neonatal gender**			
Male	27 (46.6)	30 (51.7)	0.577
Female	31 (53.4)	28 (48.3)	
**Apgar 1st,** mean ± SD	8.95 ± 0.29	8.88 ± 0.38	0.274
**Apgar 5th,** mean ± SD	9.95 ± 0.22	9.67 ± 1.36	0.129

a The p-value has been calculated between women receiving routine obstetric care versus those who underwent Ritgen’s maneuver.

Intrapartum data and maternal outcomes were analyzed (Table 3): no significant differences were found between the groups regarding labor induction, epidural analgesia, or the duration of the second stage of labor. However, a significant difference was observed in the use of oxytocin during labor, with 81% of women in the study group receiving oxytocin supplementation compared to 50% in the control group (p<0.0001).

**Table 3:** Maternal outcome and intrapartum data of the two groups of pregnant women, aged 20–40 years, divided according to the preventing perineal maneuver used in the Fondazione Policlinico Universitario Agostino Gemelli delivery unit, May 2020 – October 2021, Rome, Italy (N=116)

*Variables*	*Routine obstetric care (N=58) n (%)*	*Ritgen’s maneuver (N=58) n (%)*	*p^a^*
**Maternal comorbidities** (1 missing)	23 (39.7)	24 (42.1)	0.789
**Labor induction**	19 (32.8)	24 (41.4)	0.336
**Epidural analgesia**	44 (75.9)	50 (86.2)	0.155
**Oxytocin use**	29 (50.0)	47 (81.0)	<0.0001
**Blood loss** (mL), mean ± SD	335.34 ± 276.41	266.38 ± 246.80	0.159
**2nd Stage duration** (min), mean ± SD	68.67 ± 52.19	84.08 ± 46.26	0.095
**Occiput posterior position**	1 (2.2)	2 (3.5)	0.689
**Perineal trauma**	55 (94.8)	45 (77.6)	0.007
**Perineal trauma degree**			
1st	29 (52.7)	30 (66.7)	0.159
2nd	26 (47.3)	15 (33.3)	
3rd	0	0	
4th	0	0	

a The p-value has been calculated between women receiving routine obstetric care versus women who underwent to Ritgen’s maneuver.

In relation to study outcomes, perineal injuries were reported in 45 women (77.6%) from the study group, compared to 55 women (94.8%) in the control group (OR=0.19; 95% CI: 0.05–0.70; p=0.007). In the study group, most perineal lacerations were first-degree (66.7%), with second-degree lacerations accounting for 33.3%. In contrast, second-degree lacerations occurred in 47.3% of cases in the control group. No third- or fourth-degree lacerations were reported in both groups, so it is not possible to determine whether Ritgen’s maneuver is protective against them compared with standard perineal protection.

A further analysis was conducted to investigate the effects of Ritgen's maneuver in the case of oxytocin supplementation and epidural analgesia (Tables 4 and 5). Labor supplementation with oxytocin was used in 76 women overall (47 belonging to the study group and 29 to the control group). All women in the control group whose labor was enhanced with oxytocin reported perineal tract tears compared to 76.6% of the study group (p=0.005) (Table 4). Of the 116 women included in our study, 94 of them delivered with the use of epidural analgesia (50 belonging to the study group and 44 to the control group). Among women assisted with Ritgen’s maneuver, 24% did not experience perineal lacerations, compared to only 4.5% in the group managed with conventional obstetric practice (OR=0.15; 95% CI: 0.03–0.72; p=0.008) (Table 5).

**Table 4:** Effect of routine obstetric care versus Ritgen’s maneuver in determining perineal trauma in women exposed to treatment with oxytocin in the Fondazione Policlinico Universitario Agostino Gemelli delivery unit, May 2020 – October 2021, Rome, Italy (N=76)

*Characteristics*	*All (N=76) n (%)*	*Perineal trauma in women treated with oxytocin*		*p*
		*Yes n (%)*	*No n (%)*	
**Routine obstetric care**	29 (38.2)	29 (100)	0	**0.005**
**Ritgen’s maneuver**	47 (61.8)	36 (76.6)	11 (23.4)	

**Table 5:** Effect of routine obstetric care versus Ritgen’s maneuver in determining perineal trauma in women delivered with epidural analgesia in the Fondazione Policlinico Universitario Agostino Gemelli delivery unit, May 2020 – October 2021, Rome, Italy (N=94)

*Characteristics*	*All (N=94) n (%)*	*Perineal trauma in women delivered with epidural analgesia*		*p*
		*Yes n (%)*	*No n (%)*	
**Routine obstetric care**	44 (46.8)	42 (95.5)	2 (4.5)	**0.008**
**Ritgen’s maneuver**	50 (53.2)	38 (76.0)	12 (24)	

A logistic regression was performed to ascertain the effects of maternal age, oxytocin augmentation, epidural analgesia, Ritgen’s maneuver, labor induction, second-stage duration, and occipital position on the likelihood of having perineal trauma. The logistic regression model was statistically significant (χ^2^=18.91, p=0.004). The model explained 27.3% (Nagelkerke R^2^) of the variance in perineal trauma and correctly classified 84.6% of cases. Ritgen’s maneuver reduced the likelihood of having perineal trauma by ten times.

## DISCUSSION

Our results suggest a protective role of Ritgen’s maneuver against perineal lacerations in terms of incidence and severity of perineal trauma. Our data also confirmed its ability to reduce the perineal lesion rate in women with known intrapartum risk factors for perineal lacerations, as in the case of oxytocin supplementation or epidural analgesia. In Italy, where no national guidelines on intrapartum care are available, our results add additional and useful information regarding perineal manual protection during the second delivery stage.

The World Health Organization (WHO), in ‘Intrapartum Care for a Positive Childbirth Experience’, recommends different techniques to avoid maternal perineal trauma during the second stage of labor. Still, no conclusive evidence on which specific technique of manual perineal protection to use is available^7^. Fahami et al.^8^ were not able to demonstrate the protective role of Ritgen’s maneuver against perineal lacerations; in particular, their study conducted on 66 women suggested that women assisted with the Ritgen maneuver not only presented an increase in perineal trauma but had fewer first-degree lacerations and more second-degree lacerations than women assisted with traditional perineal protection^8^. However, no differences between the two groups were reported in third- and fourth-degree laceration rates. More recently, a systematic review conducted by Zang et al.^18^, demonstrated that the Ritgen maneuver could reduce the incidence of first-degree perineal laceration but increase the incidence of second-degree perineal laceration, but with very low-quality evidence.

The rationale for the effectiveness of Ritgen’s maneuver in protecting the perineum derives from the physiological mechanics of childbirth. During the expulsion of the fetal head, it naturally moves downward toward the perineum. The maneuver provides the necessary resistance to direct it upward, ensuring its deflection^19^. In the routinely performed MPP policy, the dominant hand exerting pressure on the perineum appears to be directed downward. This can induce excessive stretching and accelerate the deflection movement, potentially resulting in a higher frequency of posterior and anterior perineal tears^1,2,4^.

Spandrio et al.^16^ believe that, in Ritgen’s maneuver, the deflection of the fetal head should be forced with the help of the non-dominant hand; in our practice, however, the maneuver was performed in accordance with what was suggested by Habek et al.^14^: the exit of the fetal occiput was not forced in any way, but slowly accompanied with the fingers of the non-dominant hand in contact with the two fetal parietal drafts. For this reason, the pressure on the perineum from the back upwards delays the fetal head deflection, and this could explain the almost total absence of paraclitoral and paraurethral lesions and a higher incidence of intact perineum, as well as the reduction in the degree of lacerations compared to routine obstetrical practice. According to Habek et al.^14^, who conducted a retrospective study (1950–2010) in the maternity ward of a hospital in Croatia, the Ritgen maneuver is useful in the prevention of OASIS and in reducing the degree of perineal lacerations. This study has its main limitation in the lack of data regarding pregnancy, delivery, and neonatal outcomes (gestational age, maternal BMI, Apgar score, maternal pathologies, use of epidural analgesia, duration of second stage) due to inadequate use of protocols in the period from 1950 to 1990. Our study, on the other hand, although conducted on a smaller sample, has the advantage of having a complete case history due to its prospective nature; however, both studies reach the same conclusions.

Moreover, we have investigated the efficacy of Ritgen’s maneuver in the presence of certain known intrapartum risk factors for perineal trauma. According to Goh et al.^2^, oxytocin augmentation and epidural analgesia play significant roles in perineal tears. The results of our study indicate that the relative risk of perineal trauma with Ritgen’s maneuver is significantly reduced in women receiving epidural analgesia (OR=0.15; 95% CI: 0.03–0.72; p=0.008) or oxytocin supplementation (integrum perineum: 0% of the control group vs 23.4% of the study group; p=0.005). To our knowledge, no previous study has examined the correlation between Ritgen’s maneuver and perineal lacerations in these specific high-risk categories.

It is noteworthy to consider that no additional interventions were investigated in our study. Perineal protection maneuvers are just one aspect of obstetrical care and cannot solely prevent perineal damage^20^. It is worth noting that interventions during pregnancy, such as informing women about measures to reduce perineal trauma and routinely proposing perineal massage starting from the third trimester, could also be beneficial^18^. Moreover, during the second stage of labor, prevention can be enhanced through various measures such as adopting free positions or postures, creating an intimate and protected environment, respecting the timing of pushing, and utilizing warm compresses^7^. For all these reasons, additional studies that take into account these variables are warranted.

In addition, the literature suggests an association between perineal tears and neonatal weight^2^. Although the difference was not statistically significant between the two groups, women assisted with Ritgen’s maneuver gave birth to infants weighing between the 25th and 50th percentiles to a greater extent than the control group (48.3% vs 31.0%, p=0.07); conversely, in the group in which conventional perineal protection was performed, there was a greater prevalence of infants weighing between the 50th and 75th percentiles. This could imply that the lower, although not significant, average birth weight played a role in the reduced incidence of perineal lacerations in the study group.

### Strengths and limitations

Including complete medical histories for all participants ensures robust data integrity. Moreover, due to the prospective nature of the study, the researcher was always present at the time of delivery for the intervention group. However, this was not possible for the control group. These, therefore, represent further limitations of our study. In our study, only one midwife was assigned to perform the Ritgen maneuver to avoid bias related to technique and professional experience; however, in the control group, we included all women assisted according to conventional practice without considering which operator had performed the maneuver. This study was conducted in a high-resource setting with free access to universal healthcare; therefore, the findings are likely generalizable to similar healthcare systems and perinatal care access.

## CONCLUSIONS

Our research suggests that Ritgen’s maneuver is important in preventing perineal lacerations and reducing their degree. Its efficacy has also been demonstrated in the presence of some known risk factors for perineal trauma, such as epidural analgesia or oxytocin augmentation. The diversity of its execution across the existing studies may explain some of the differences in perineal lacerations observed by other authors. Thus, the literature is still not univocal in recommending an effective maneuver to protect the perineum to reduce the incidence of obstetric perineal trauma. Midwives play a key role in the prevention of perineal injury, and, often, the techniques they use vary significantly, as do the motivations behind them. Our study proposes a valid and appropriate perineal protection maneuver in childbirth care to reduce the incidence and degree of perineal tears, thus suggesting a useful tool for clinical practice. Our study stressed the importance of conducting further research to identify if Ritgen’s maneuver may prevent these adverse long-term perineal outcomes. Further research should also be conducted to establish long-term outcomes, such as perineal site pain during the puerperium period and uro-gynecologic dysfunction.

## Data Availability

The data supporting this research are available from the authors on reasonable request.
